# A Brief History of Pacemaking

**DOI:** 10.3389/fphys.2019.01599

**Published:** 2020-01-22

**Authors:** Dario DiFrancesco

**Affiliations:** Department of Biosciences, University of Milano, IBF-CNR University of Milano Unit, Milan, Italy

**Keywords:** pacemaker, funny current, I_f_ current, HCN channels, cardiac rate, ivabradine, HCN channelopathies

## Abstract

Cardiac pacemaking is a most fundamental cardiac function, thoroughly investigated for decades with a multiscale approach at organ, tissue, cell and molecular levels, to clarify the basic mechanisms underlying generation and control of cardiac rhythm. Understanding the processes involved in pacemaker activity is of paramount importance for a basic physiological knowledge, but also as a way to reveal details of pathological dysfunctions useful in the perspective of a therapeutic approach. Among the mechanisms involved in pacemaking, the “funny” (I_f_) current has properties most specifically fitting the requirements for generation and control of repetitive activity, and has consequently received the most attention in studies of the pacemaker function. Present knowledge of the basic mechanisms of pacemaking and the properties of funny channels has led to important developments of clinical relevance. These include: (1) the successful development of heart rate-reducing agents, such as ivabradine, able to control cardiac rhythm and useful in the treatment of diseases such as coronary artery disease, heart failure and tachyarrhythmias; (2) the understanding of the genetic basis of disorders of cardiac rhythm caused by HCN channelopathies; (3) the design of strategies to implement biological pacemakers based on transfer of HCN channels or of stem cell-derived pacemaker cells expressing I_f_, with the ultimate goal to replace electronic devices. In this review, I will give a brief historical account of the discovery of the funny current and the development of the concept of I_f_-based pacemaking, in the context of a wider, more complex model of cardiac rhythmic function.

## Early Membrane-Limited Pacemaker Theories

Early theories of pacemaking (Noble, 1960) were based on the assumption of the “decay” of a K^+^ current, a concept originally proposed in 1951 by Silvio Weidmann on the basis of conductance measurements during diastolic depolarization (DD) in Purkinje fibers (Weidmann, 1951).

The idea received strong support in 1968 with the recording in Purkinje fibers of a pure K current (I_K2_) with properties fitting perfectly those required for a “pacemaker” current (Noble and Tsien, 1968). This, and a bulk of experimental and theoretical data collected since its original proposal crystallized the K^+^ current-decay hypothesis for almost 30 years, with no one having the slightest doubt about its validity.

## Discovery of an Intruder: the Funny Current

In 1979 novel, apparently confounding evidence appeared in the shape of a new ion current recorded from rabbit SAN preparations (Brown et al., 1979). The “funny” (I_f_) current was so baptized because it displayed unusual properties, such as a previously unreported nature of *inward* current activated on *hyperpolarization*. This feature gave it the ability to start the DD following an action potential (AP), hence to *generate* spontaneous activity, fitting nicely the requirement of a perfect pacemaker current. Furthermore, we realized that the acceleration caused by adrenaline was due to an increased DD slope, and asked whether I_f_ could be responsible; to answer this we simply perfused with adrenaline a preparation and watched what happened to I_f_. What a beautiful experiment! I_f_ increased in agreement with the increased steepness of DD. So not only had we discovered a novel mechanism for pacemaker generation, we also had found that it controlled cardiac rate!

The finding of I_f_ introduced a novel concept in the pacemaking scenario: pacemaking depended upon activation of an inward current at the termination of an AP. This notion was exactly the opposite of the classical one based on deactivation of an outward current. Could we really have two distinct pacemaker mechanisms, one in the SAN and one in the Purkinje fibers?

## Potassium Ion Accumulation/Depletion Phenomena Can Make Voltage-Clamping a Deceitful Affair

New theories revolutionizing established concepts often depend on new techniques.

A new technique developed in the mid-late 70’s to voltage-clamp mammalian SAN, the natural cardiac pacemaker region, rather than Purkinje fibers (Noma and Irisawa, 1976), indeed made a big difference. Voltage-clamp of Purkinje fibers, but not of SAN preparations, had in fact a serious and deceitful limitation, one that could make, as discussed below, an inward current look like an outward current: extracellular depletion of K^+^ ions.

Anatomy matters. Purkinje fibers are composed of large, tightly packed cells electrically connected along the longitudinal, but not the transverse direction; restricted extracellular spaces, called clefts, separate each cell from the neighboring ones. SAN cells, on the other hand, are loosely connected to each other, without a preferential direction of signal propagation, and are not surrounded by clefts.

The K^+^-ionic nature of the I_K2_ current had been based on evidence for a reversal potential near the K^+^ equilibrium, as measured by applying large negative steps (Noble and Tsien, 1968). A few years later, however, several studies showed the presence of K^+^ accumulation/depletion phenomena in Purkinje fibers (Attwell et al., 1979; Brown et al., 1980; DiFrancesco and Noble, 1980). In fact, stimulated by premonitory curiosity, Carlos Ojeda, Mitsuyoshi Ohba and myself had found that even the measurement of the I_K2_ reversal potential was distorted by cleft K^+^ depletion (DiFrancesco et al., 1979).

Driven by intuitive curiosity, further studies highlighted the striking resemblances between I_f_ and I_K2_ (Brown and DiFrancesco, 1980; DiFrancesco and Ojeda, 1980). Yet the Purkinje fiber I_K2_ reversed at negative voltages, but I_f_ did not. So where did the solution to the puzzle lay?

## I_K2_ Reinterpretation: a Unifying Model of Pacemaking

The idea that the best described and most famous cardiac current could have been wrongly interpreted and described, in a way that had misled the whole of the scientific community for such a long time, was not to be admitted easily. A systematic study of the properties of I_K2_ eventually led to the, by then half-expected, shocking result: I_K2_ was not an outward, but an inward current! (DiFrancesco, 1981). This was shown by three lines of evidence: block by Cs^+^, conductance measurement during negative steps, and the most impressive bit of evidence: by blocking K^+^-depletion, barium ions blocked a large, inward-decreasing component which overlapped the inward increasing I_f_, in such a way as to determine a “fake” reversal.

In other words, the I_K2_ current was in fact a camouflaged I_f_! The apparent reversal near the K^+^- equilibrium potential was the result of two distinct overlapping events: activation of I_f_ and an inward-decaying K^+^ current due to K^+^ depletion in extracellular clefts. The nature of the pacemaker process in cardiac cells had therefore baffled the scientific community for over a decade, and the I_K_-decay hypothesis had remained undisputed for almost three decades.

Feeling uneasily as a young physiologist who was challenging a central dogma of cardiac physiology, established decades before, fully accepted by the scientific community and supported by world-leading labs, I hardly knew how to best communicate this to the scientific community. I eventually decided to share this knowledge with Denis Noble, and called him over the telephone to reveal my findings. This further strengthened our collaborative relation and resulted a few years later in a numerical model published by Philosophical Transactions of the Royal Society (DiFrancesco and Noble, 1985). The model incorporated the I_f_ current and other new data, and accounted for essentially all previous experimental data in Purkinje fibers. It was a landmark work in the field of numerical reconstruction and laid the basis for future numerical modeling. It received an important recognition when in 2015 the Royal Society (London) celebrated its 350 year-anniversary and selected the 33 most influential papers published in 350 years by the Philosophical Transactions. Along with papers by Newton, Faraday, Joule, Maxwell, Turing, Medawar, and other giants, Denis and I found that our 1985 model paper had also been listed, to our great honor and gratification.

The discovery of I_f_ and the reinterpretation of I_K2_ showed that the mechanism generating pacemaker activity was the same in different myocytes and represented a unifying theory of pacemaking.

## The I_F_ Properties Are Well Suited for a Pacemaker Current

The I_f_ discovery in 1979 and the reinterpretation of I_K2_ in 1981 paved the way for a large number of worldwide studies which, in the course of about four decades, investigated the properties of this current.

This part has been amply covered by many review articles (DiFrancesco, 1985, 1987, 1993, 1995, 2006, 2010; DiFrancesco and Camm, 2004; Baruscotti et al., 2005; Barbuti et al., 2007; Bucchi et al., 2012; DiFrancesco and Noble, 2012) and only the most relevant findings are mentioned here.

Kinetics and ionic nature of the current were first investigated and revealed that the current activates slowly, without inactivation, upon hyperpolarization to the diastolic range of voltages (DiFrancesco, 1981; DiFrancesco et al., 1986). Its mixed Na^+^ and K^+^ ionic nature, another unusual property for a voltage-dependent channel, ensures that I_f_ is inward in its activation range (DiFrancesco, 1981).

These properties are well designed for a pacemaker current, since slow activation of an inward current at the termination of an action potential, when the voltage enters the pacemaker range, is bound to contribute to a depolarizing process such as diastolic depolarization.

I_f_ single-channel activity was first recorded in 1986 using an experimentally demanding protocol, involving the use of two pipettes on the same cell to increase single-channel resolution. This was necessary because of what would eventually result one of the smallest ever recorded single channels, with a conductance of about 1 pS (DiFrancesco, 1986). Because channel opening during activity is a stochastic process, low conductance of f-channels is useful to avoid too large Heart Rate Variability (HRV).

Also essential was the finding that not only is I_f_ activated by adrenergic stimulation, it is also strongly inhibited by muscarinic stimulation (DiFrancesco and Tromba, 1988a, b). This endowed the I_f_ current with a significant physiological role not only in the process of generation of pacemaker activity, but also in the autonomic regulation of cardiac rate.

The relevance of muscarinic I_f_ modulation was further strengthened by the finding that low ACh concentrations slow heart rate by I_f_ inhibition and not, as previously believed, by activation of an ACh-activated K^+^ current (I_KACh_) (DiFrancesco et al., 1989). Thus, normal vagal tone keeps a relatively low heart rate at rest by means of a basal ACh-induced I_f_ inhibition.

How did autonomic transmitters modulate I_f_? Experimental data indicated the presence of a shift of the activation curve (positive with adrenergic, negative with vagal stimulation), with no change of fully-activated currents. Recording of I_f_ from giant inside-out patches containing hundreds of channels, another demanding protocol, led to the discovery that f-channels are directly activated by binding of intracellular cAMP, via a positive shift of the activation curve (DiFrancesco and Tortora, 1991). Other known channels had this property (typically the cGMP-activated channels of the retina), but f-channels had the unique feature of being *dually* activated by both voltage (hyperpolarization) and cAMP. These data completed the full range of processes involved in the I_f_-mediated autonomic rate modulation.

At the turn of the century/millennium, a new era developed for the funny current, following the cloning of the HCN (hyperpolarization-activated, cyclic nucleotide-gated) channel family (Gauß et al., 1998; Santoro et al., 1998; Vaccari et al., 1999). Of the 4 isoforms cloned (HCN1-4), the HCN4 isoform is the most highly expressed in pacemaker tissue.

Cloning of HCN channels allowed investigating the molecular basis of the properties of I_f_ originally discovered decades before. More recently, the molecular structure first of portions (Saponaro et al., 2014) and finally of the entire α-subunit of HCN channels [HCN1 (Lee and MacKinnon, 2017)] has been resolved. It is quite satisfactory to find that all the features described some 40 years before, such as activation on hyperpolarization, cAMP-dependent activation, mixed Na^+^/K^+^ permeability, have eventually found a perfectly fitting molecular interpretation.

## Practical Applications of the Functional Properties of Funny/Hcn4 Channels

The discovery of I_f_ was initially important as a basic concept in cardiac (and non-cardiac) physiology, but progressively more detailed knowledge of its role in pacemaker generation and cardiac rate control has led more recently to the development of practical applications of clinical relevance.

An important application concerns the pharmacological control of heart rate. Pharmacological research has been long seeking for substances able to slow heart rate specifically, without the side effects of β-blockers and Ca^++^ antagonists. Several “heart rate-inhibiting” substances have been developed to this aim. Ivabradine, the only such drug having reached the market, is a selective f-channel blocker which slows heart rate with little or no cardiovascular side effects, now successfully used in the therapy of Coronary Artery Disease and heart failure (DiFrancesco and Camm, 2004; DiFrancesco and Borer, 2007). Ivabradine directly validates the important contribution of I_f_ to control of DD and heart rate.

A second application concerns the genetics of arrhythmias. As expected from the I_f_ role in pacemaking, several HCN4 mutations have been identified in patients with alterations of cardiac rhythm (DiFrancesco, 2013). Arrhythmias associated with HCN4 mutations can be complex, but the majority of reported mutations are loss-of-function and are associated with bradycardia, in agreement with the funny channel involvement in rate control. Interestingly, the only tachyarrhythmia-associated HCN4 mutation found is gain-of-function, which again fits perfectly the I_f_ role in pacemaking (Baruscotti et al., 2015). Thus HCN4 mutation-linked arrhythmias, too, provide evidence confirming the pacemaking role of I_f_.

A final example of clinically relevant application concerns the development of biological pacemakers. Several attempts have shown that *in situ* delivery of funny channels to defective cardiac muscle by gene- or cell-based methods can be employed in the attempt to develop biological pacemakers, with the aim to replace electronic devices. Exhaustive review work covers this important subject (Robinson et al., 2006; Rosen et al., 2011; Chauveau et al., 2014).

The underlying idea is that f-channels can transfer their “pacemaking” ability, so that silent cells made to express f-channels become spontaneously active. Early studies showed for example that HCN2 transfer to neonatal ventricular myocytes accelerates DD and spontaneous rate (Qu et al., 2001), and other attempts used adenoviral-mediated HCN infection, HCN-channel expressing mesenchymal stem cells, fusion between HCN1-expressing fibroblasts and myocytes (Qu et al., 2003; Plotnikov et al., 2004; Potapova et al., 2004; Bucchi et al., 2006; Kashiwakura et al., 2006; Cho et al., 2007; Plotnikov et al., 2008). Cardiomyocytes derived from embryonic stem cells, known to express I_f_ (Barbuti et al., 2009), have also been adopted to pace cultured cardiomyocytes and *in vivo* hearts when properly grafted (Kehat et al., 2004; Xue et al., 2005; Ionta et al., 2015; Protze et al., 2017). HCN-expressing induced Pluripotent Stem Cell (iPSC)-derived cardiomyocytes have also been used to successfully pace immunosuppressed dog hearts (Chauveau et al., 2017). Though still mainly as proof-of-concept, these data show that HCN transfer can be a viable method to generate biological pacemakers.

## A New Contender: the Ca Clock

Pacemaking involves a large number of events, both at the membrane and inside the cell, which are tuned together to achieve reliable generation and modulation of spontaneous rhythm. This does not exclude that any single mechanism in this network may have a specific function.

For example, the relevance of I_f_ activation does not imply that no K+-flow occurs during diastole; indeed the I_Kr_ current has been shown to contribute importantly to pacemaking, while having at the same time a major role in repolarization (Clark et al., 2004).

As discussed above, the properties of the funny current are fit for a mechanism contributing substantially to the DD process, generation of cyclic repetitive electrical activity and, ultimately, control of cardiac rate. Clearly, however, all participating mechanisms, not only funny channels, cycle rhythmically during pacemaker activity, and an obvious question arises if other mechanisms with a *specific* role in generating pacemaker activity exist.

Among the important cycling elements are Ca^++^ ions. Ca^++^ ions are released cyclically from the Sarcoplasmic Reticulum (SR) according to a Ca^++^-dependent Ca^++^-release mechanism, leading to rhythmic fluctuations of the intracellular Ca^++^ concentration, a mechanism directly responsible for mechanical contraction.

In rabbit SAN cells, Lakatta and collaborators reported that the Ca^++^ concentration increased in sub-sarcolemmal spaces due to opening of Ryanodine Receptors (RyR) which occurred rhythmically during the final fraction of the diastolic depolarization, just before AP upstroke (Bogdanov et al., 2001; Vinogradova et al., 2002, 2004). Since these Local Calcium Releases (LCRs) could generate Ca^++^ waves via Ca^++^-dependent Ca^++^-release, thus activating the Na-Ca exchange (NCX) and associated inward depolarizing current, they contributed to accelerate the late phase of diastolic depolarization. This set of events was termed “Ca^++^ clock” and proposed to represent an alternative pacemaker mechanism.

Several studies have investigated the properties of the “Ca^++^ clock” mechanism in order to address its functional role in generating spontaneous activity in pacemaker cells.

Lakatta’s group has reported that LCRs are roughly periodic with a rate similar to the SAN beating rate, and can be recorded in permeabilized cells and under V-clamp (Vinogradova et al., 2004). Based on this and other evidence, the Ca^++^ clock model predicts that pacemaker repetitive activity is not caused by “membrane” processes but rather by intracellular Ca^++^ cycling coupled to the NCX current, and that late diastolic Ca^++^ releases are an obligatory rhythmic process in pacemaking [Lakatta in Lakatta and DiFrancesco (2009)].

Whether the Ca^++^ clock is a pacemaking mechanism and how this relates to the I_f_ –based “membrane clock” mechanism are debated questions (DiFrancesco and Robinson, 2002; Lakatta et al., 2003; Lipsius and Bers, 2003; Lakatta and DiFrancesco, 2009; Robinson, 2011).

A first obvious difficulty for the “Ca^++^ clock” hypothesis is that it completely lacks any degree of specificity for pacemaker tissue. Local Ca^++^ releases and more in general Ca^++^ transients are involved in mechanical contraction, which obviously occurs in all cardiac myocytes. Funny channels, on the other hand, are expressed physiologically only in the SAN and conduction tissue, i.e., only in the myocytes able to beat spontaneously.

There are other problems with the assumption that the Ca^++^ clock is a main determinant of the timing of cardiac rhythm [see DiFrancesco in Lakatta and DiFrancesco (2009), DiFrancesco and Noble (2012)].

For example, disruption of intracellular Ca^++^ dynamics obtained by intracellular perfusion with the Ca^++^ chelator BAPTA totally removes Ca^++^ transients, but leaves repetitive electrical activity to continue undisturbed for tens of second in a single SAN cell (Himeno et al., 2011). This cannot be reconciled with the assumption that LCRs are an obligatory pacing process.

Also, a recent study of intracellular Ca^++^ transients in pacemaker myocytes from mice lacking the L-type Ca^++^ channels Cav1.3 has shown that in these myocytes, the rate of LCRs is greatly reduced; further, in the presence of β-adrenergic stimulation, residual LCRs are asynchronous and not concentrated in the last fraction of the diastolic depolarization (Torrente et al., 2016). These data agree with the hypothesis that LCRs are caused by Ca^++^ entry through Cav1.3 channels typical of pacemaker cells, in contrast with the Ca^++^-clock assumption that they are caused by spontaneous, rhythmic release from ryanodine receptors.

As mentioned, pacemaking is a composite phenomenon and any process directly or indirectly modifying the diastolic depolarization will affect rate. Furthermore, several recent studies have highlighted new complexities in the pacemaker scenario. While a full discussion of these studies is not presented here, it is important to stress that pacemaking is also modulated by various enzymes (DiFrancesco, 2019; Lin et al., 2019) and mechanical stretch (Quinn and Kohl, 2012). Further, investigations of genetically modified mouse models have shown that Ca_*v*_1.3 channels, specifically expressed in pacemaker tissue, contribute directly to pacemaker depolarization (Mesirca et al., 2015), and underlie a persistent Na current (Toyoda et al., 2017), identifiable with the Na-background current hypothesized in early numerical reconstructions.

Also importantly, GIRK4 channel inactivation has been shown to rescue bradyarrhythmias of HCN4-deficient mice (Mesirca et al., 2014), highlighting a previously unsuspected role of I_KACh_ in counterbalancing I_f_ changes. Complexities in the autonomic modulation of rate have also been described by use of genetically modified mice (Kozasa et al., 2018). These studies suggest that funny channels act to limit excessive bradycardia caused by potent parasympathetic activity.

Existing pacemaker models will clearly need to be extended to comprise this fuller set of novel acquisitions.

## Conclusion

The initial major impact of the discovery of the funny current was that it revolutionized the old, well-established, worldwide-accepted model of the origin of pacemaking and provided a rational explanation of how pacemaker activity is generated and controlled by the autonomic nervous system.

The I_f_ -based pacemaking model has since advanced from the original, basic concept mostly relevant to textbook cardiovascular physiology, to a practical concept that can be exploited to generate many useful clinical applications valuable for the development of new therapies.

Pacemaking is a complex mechanism and needs the co-operation of many elementary processes, each of which is unable, by itself, to generate a robust and secure action potential at a stable rate. Still, each of these mechanisms has a specific role. In agreement with their unique functioning role, funny channels are specifically expressed, under physiological conditions, in pacing cells only. Local Calcium Releases occurring in late diastole in subsarcolemmal spaces of pacemaker cells appear to depend upon entry of Ca^++^ ions through Cav1.3 channels and may thus represent a mechanism apt to boost the action potential upstroke, rather than autonomously timing pacemaker rate.

## Author Contributions

The author confirms being the sole contributor of this work and has approved it for publication.

## Conflict of Interest

The authors declare that the research was conducted in the absence of any commercial or financial relationships that could be construed as a potential conflict of interest.
